# Lower Urinary Tract Urological Abnormalities and Urodynamic Findings of Physiological Urinary Incontinence Versus Non-mono Symptomatic Nocturnal Enuresis in Children

**DOI:** 10.5812/numonthly.15360

**Published:** 2014-03-10

**Authors:** Mitra Naseri

**Affiliations:** 1 Pediatric Nephrology Department, Dr. Sheikh Children Hospital, Mashhad University of Medical Sciences, Tehran, IR Iran

**Keywords:** Urinary Tract Anomalies, Physiological Urinary Incontinence, Nocturnal Enuresis, Children

## Abstract

**Background:**

Although 98% of children attain daytime bladder control by three years of age, urinary incontinence is regarded physiological up to the fifth year of life.

**Objectives:**

This study aimed to assess whether lower urinary tract urological abnormalities and abnormal urodynamic findings are infrequent in children with physiological urinary incontinence in contrast to those with non-monosymptomatic nocturnal enuresis (NMNE).

**Patients and Methods:**

During a three-year period (2007-2009), 66 neurologically normal children including 51 children (34 girls, 17 boys) older than five years of age with NMNE and intermittent daytime incontinence, and 15 children with physiological urinary incontinence (eight girls and seven boys) aged four to five years of age without any known urological abnormalities were enrolled in the study. Patients with neurologic deficits or known urological anomalies were excluded from the study. Kidney-bladder ultrasonography, voiding cystourethrography (VCUG), and urodynamic studies were performed to evaluate the anatomy of urinary tract and bladder function.

**Results:**

Urinary tract infection was found in 23 (34.8%) children, 17 (33.3%) and 6 (40%) patients with NMNE and physiological urinary incontinence, respectively. Out of 48 patients who underwent VCUG, vesicoureteral reflux (VUR) was found in seven and eight children younger and older than five years of age, respectively. Abnormal urodynamic findings were reported in 5 (62.5%) of eight children younger than five-year-old, and 14 (63.6%) of 22 patients older than 5-year-old.

**Conclusions:**

VUR might be more frequent in children with physiological urinary incontinence than the normal population, and might be as common as NMNE with intermittent daytime incontinence.

## 1. Background

The time of attaining urinary continence is variable among different individuals. The range of normal continence development is very wide. Urinary incontinence is regarded physiologic up to the fifth year of life. Many children experience physiological urinary incontinence beyond their fifth year of life and are considered as late developers (1). By the age of three to four years, most children have an adult pattern of urinary control and are dry both day and night (2, 3). Normal daytime and nighttime control of bladder function is attained between two to three and three to seven years of age, respectively (4, 5). Twenty six percent of children attain daytime continence by the age of 24 months, 52.5% by 27 months of age, 85% by 30 months of age, and 98% by 3 years of age (6). The age of daytime bladder control might vary from 9 months to 5.25 years (mean of 2.4 years of age), and it is attained slightly earlier in females (7). The etiologies of urinary incontinence can be organic and functional (8, 9). In contrast to adults in whom the incontinence usually heralds a pathologic condition, incontinence in children is usually functional and does not require invasive studies (9). Voluntary control over the lower urinary tract needs complex interactions between autonomic and somatic efferent pathways (10). Less than 10% of healthy neonates and infants have cystometrically detectable detrusor overactivity and detrusor-sphincter discoordination (DSD). Owing to DSD, voiding during the first years of life is often incomplete, however, it disappears when bladder control is attained (11).

## 2. Objectives

We conducted a pilot study to define whether lower urinary tract (LUT) urological abnormalities, mainly vesicoureteral reflux (VUR) and posterior urethral valve (PUV), and abnormal urodynamic findings are common in children with physiological urinary incontinence (UI). In addition we aimed to compare the prevalence rate of these abnormalities in children with non-mono symptomatic nocturnal enuresis (NMNE) associated with intermittent daytime incontinence.

## 3. Patients and Methods

During a three-year period (2007-2009), children older than four-year-old who were referred to a tertiary academic health center due to day and night wetting were screened. In the first step of the study, those with known urological disorders (especially VUR and PUV) or neurologic deficits (myelodysplasia, spinal injury or surgery, cerebral palsy, or mentally retarded children) were excluded from the study. Excluded patients included seven children with myelomeningocele and three children with spinal cord involvement by malignant lesions (two children with pelvic malignancies and another one with lymphoma). In children older than five years of age, the international children continence society (ICCS) criteria were used to define LUT symptoms (12). UI was defined as the continuous or intermittent uncontrollable leakage of urine. Continuous incontinence is applicable for all ages whereas intermittent incontinence is applicable only to the children with at least five years of age (12). The term of physiological UI was used for children younger than five years who wetted themselves day and night (1). Nocturnal enuresis was defined as the incontinence in discrete episodes during sleep in children older than 5 years of age; NMNE was considered as nocturnal enuresis associated with daytime symptoms (12). Association of bowel symptoms with LUT symptoms was emphasized. Constipation was considered as delay or difficulty in defecation that was present for two or more weeks sufficient to cause distress. Encopresis was defined as voluntary and involuntary passage of feces in the inappropriate places in a child with four years of age or older without any known organic etiology (12). The included patients were allocated into two groups:

Group 1 included 51 children older than five years of age with NMNE who had intermittent daytime incontinence.

Group 2 included 15 children aged four to five years of age with physiological UI. The number of late developer referred to clinic during the study was low, and the study in this group was performed as a pilot study. The study was funded by a research grant from Mashhad University of Medical Sciences and approved by local ethic committee. Oral consents were obtained from patients or their parents. A checklist was designed and details of personal history, voiding, bowel symptoms, and abnormal findings on physical examination (with special attention to genital, abdominal, and lumbosacral spines) were recorded. The eligible patients underwent urine analysis, urine culture (by midstream method), and three dimensional kidney bladder ultrasonography (US). Forty-eight of included children accepted to undergo voiding cystourethrography (VCUG) and amongst them, 35 participants accepted to undergo uroflowmetry (Chart A). Uroflowmetry is a noninvasive urodynamic study (UDS) which assesses voiding function by measuring the rate of voided urine flow (13). Uroflowmetry and VCUG were performed using standardized techniques (12, 14-16). Cystometrography (CMG) and pelvic floor electromyography (EMG) were performed in children with of abnormal findings in uroflowmetry, abnormal bladder US findings including bladder wall thickness (BWT), decreased or increased bladder volume (BV), or post voiding urinary residual volume (PVURV) of more than 15 mL. Frequency-volume charts were not obtained since patients and their parents did not cooperate appropriately to collect and measure numbers and volumes of urine samples.

Renal scintigram (TC99-DMSA scan) was performed in patients who were at greater risk for renal cortical damage including those with VUR and abnormal US findings such as small sized kidney or evidence of renal scars.

### 3.1. Definitions Used in the Study

1. Urinary tract infection (UTI) was defined as positive urine culture (growing of one microorganism with colony count ≥ 10 ^5^) in symptomatic patients.

2. Terms used for interpretation of bladder US:

A. Changes of bladder volumes (BV) including increased and decreased BV defined by matching the calculated volume with normal volume for different ages (17).

B. Bladder wall thickness (BWT) ≥ 3 mm in filled bladder was considered as increased.

C. PVRUV of more than 15 mL was defined as abnormal finding (12, 18).

3. Normal uroflowmetry was defined as bell shaped curve and abnormal uroflowmetry applied for tower, plateau, staccato, and interrupted patterns of voiding (19). Pediatric uroflowmetry curves interpretation is incompletely standardized (19). ICCS proposed standardizing the terminology for such abnormal curves and classified them as bell (normal), tower, plateau, staccato, and interrupted patterns. Plateau curve has a low amplitude and rather even flow. Staccato pattern has at least one dip greater than the square root of the maximal flow rate. Tower-shaped curve is a high amplitude curve of short duration. Interrupted curve is defined as the curve reaching the baseline during voiding (12).

4. Abnormal VCUG finding (20):

A. Spinning top deformity: bladder neck opening with bulging to posterior urethra up to contracted external sphincter.

B. Widening of bladder neck: Open bladder neck during the bladder filling phase.

C. Bladder diverticula: Herniation of vesical mucosa through a defect in the muscular wall of the bladder.

5. Terms used for interpretation of EMG and CMG:

A. The volume in the filling phase at which the patient felt first desire to void (FDV) was defined as bladder capacity. FDV occurs as patient approaches functional bladder capacity (12, 21).

B. Measured capacity less than 65% of the calculated value for age was defined as small capacity and measured volume more than 150% of the calculated value for age was considered as large (high) capacity bladder (1, 22).

C. Detrusor overactivity (detrusor hyperactivity) was defined as involuntary detrusor contractions during the filling phase, involving a detrusor pressure increase of more than 15 centimeters of water above the baseline value (12).

D. Detrusor underactivity was defined as a contraction of decreased strength resulting in prolonged bladder emptying and/or failure to achieve complete bladder emptying (12).

E. Overactive bladder (OAB) was defined as involuntary detrusor contractions, small bladder capacity, and urethral instability (12, 14).

6. Cortical uptake defects on renal scintigram were defined as cortical renal scars (23).

#### 3.1.1. Methods Used for Measuring the Bladder Indices

For defining the bladder volume, the distance was measured from longitudinal and transverse images through the largest portion of bladder in each plane. Linear measurements from longitudinal images were height (H) and depth (DL). The width was measured from transverse images. Bladder volume was measured in filled bladder when the child felt a need to empty the bladder.

Bladder wall thickness was measured on longitudinal and transverse images in bladder floor posterior to trigone in filled bladder and matched with age; PVRUV was recorded by measuring the bladder volume after emptying the bladder (17).

### 3.2. Methods Used in UDS

Uroflowmetry was performed as the first step of urodynamic evaluation. For uroflowmetry testing, children were asked to wait until they felt at least a strong desire to void. It was performed in three consecutive times. CMG was performed as a conventional method involving dual-lumen urodynamic catheter and a rectal catheter. The bladder was filled slowly with saline warmed to 37°C in supine position (12). Intravesical and intra-abdominal pressures were recorded simultaneously and detrusor pressure was derived. Abdominal pressure was estimated from rectal catheter. All UDS were performed in the awake state. Pelvic floor EMG was performed by inserting skin electrodes. Bladder capacity was measured by the Koff formula as follows: Volume (mL) = ((Age + 2) × 30) (14)

Clinical details, imaging, and urodynamic findings between the two groups were compared by Chi square and Fisher exact tests using SPSS Inc version, and P-value ≤ 0.05 was regarded as statistically significant.

## 4. Results

There were 34 (66.7%) girls and 17 (33.3%) boys, and 8 (53.3%) girls and 7 boys (46.7%) in groups 1 and 2, respectively. Association of UI with constipation and encopresis was more strong in children of group 2 than in children of group 1 (P = 0.743 and P = 0.749, respectively). These symptoms were reported in four and five (one-third of patients) patients with physiological UI, and 11 and 13 (about 25%) patients with NMNE, respectively (Table 1). 

**Table 1.** Urinary and Bowel Symptoms in Patients With NMNE

Symptom	No. (%)
**Increased voiding frequency **	13 (24.5)
**Straining**	1 (2)
**Constipation**	11 (21.6)
**Encopresis **	13 (4.5)
**Wetting during nap**	15 (29.4)
**Urge incontinence **	18(35.3)
**Holding maneuvers **	7 (13.7)
**Dribbling **	3 (5.9)
**Giggle incontinence **	1 (2)
**Decreased voiding frequency **	3 (5.9)
**Encopresis + constipation **	6 (11.8)
**Urge incontinence + increased voiding frequency**	3 (5.9)
**Intermittency**	3 (5.9)
**Total**	51

In the first evaluation, 17 and six patients in groups 1 and 2 had urinary tract infection (UTI), respectively (P = 0.659). In group 1, 8 (24.2 %) out of 33 children who underwent VCUG had VUR that had involved ten kidney-ureter units (KUUS), whereas VUR was reported in 7 (46.7%) of 15 patients of group 2 with involvement of eight KUUS. The grades of VUR were 1 (2), 3 (5), 4 (2) and 5 (1) in group 1, and 2 (1), 3 (3), 4 (2) and 5 (2) in group 2. We did not find any significant differences regarding the prevalence of VUR between study groups (P = 0.508). No urethral abnormality was reported in children who underwent VCUG.

Table 2 compares the results of bladder US and VCUG in the participants. Uroflowmetry curves in group 2 revealed bell shape or normal pattern, plateau pattern, and staccato voiding in five, two, and four patients, respectively while in group 1 these patterns were reported in ten, eight, and one children, respectively. Two patients with NMNE had tower pattern, and the results were not reliable in three children. Eight children with physiological UI (about 50% of patients) and 22 (43%) of 51 children with NMNE met the criteria to undergo EMG and CMG. Based on the results of UDS, patients were divided into five groups (Table 3). As Table 3 shows, the main abnormal UDS finding was OAB, which was reported in 16 (53.3%) out of 30 patients.

**Table 2.** Bladder US and VCUG Findings in Our Series ^a^

Findings of VCUG	Children With Physiological UI	Children With NMNE
**Normal**	6 (40)	13 (39.4)
**Irregularity of bladder wall**	3 (20)	6 (18.2)
**Vertical bladder**	3 (20)	1 (3)
**Widening of bladder neck**	2 (13.3)	5 (15.2)
**Unilateral VUR**	6 (40)	6 (18.2)
**Bilateral VUR**	1 ( 6.7)	3 (9)
**Spinning top deformity**	2 ( 13.3 )	1 (3)
**Enlarged bladder**	0	0
**PUV**	0	0
**Bladder Diverticulum**	0	2 (6)
**Total**	15 (100)	33 (100)
**Bladder US Findings**	Children With Physiological UI	Children With NMNE
**Normal**	5 (35.7)	6 (11.8)
**Increased bladder wall thickness**	8 (57.1 )	37 (72.5)
**Post voiding urinary residue**	2 (14.3)	8 (15.7)
**Decreased bladder volume**	0	3 (9.5)
**Increased bladder volume**	1 (9)	0
**Widening of bladder neck**	0	1 (2)
**Total**	14 (100)	51 (100)

^a^ Data are presented in No. (%).

**Table 3.** Urodynamic Studies Results in Patients ^a, b^

Findings of UDS	Group 1	Group 2
**Group 1 (normal UDS)**	3 (13.6)	3 (37.5)
**Group 2 (OAB)**	3 (13.6)	1 (12.5)
**Group 3 (OAB + detrusor hyper activity)**	9 (40.9)	3 (37.5)
**Group 4 (detrusor hyper activity)**	2 (9)	1 (12.5)
**Group 5 (under active bladder)**	0	0
**Unreportable**	5 (22.7)	0
**Total**	22 (100)	8

^a^ Abbreviation: UDS, urodynamic studies.

^b^ Data are presented in No. (%).

Renal scintigram was performed in 16 patients (11 and five patients in groups 1 and 2, respectively) and revealed renal cortical uptake defect (renal scar) in ten participants including four children with physiological UI and six with NMNE. Eight out of ten children with renal cortical scar had VUR (Table 4).

**Table 4.** Clinical, Imaging and Urodynamic Characteristics of Patients With VUR ^a^

Gender	Age, y	Abnormal Bowel Symptoms	History of UTI	Family History of Enuresis	Bladder US	Kidney US	VCUG Findings	Tc99-MSA Findings	Bladder Function Based on UDS
**Male**	4	Negative	Negative	Positive	Normal	Right hydrouretero-nephrosis	VUR grade 4 on RK	Stage 1 of renal scar in RK	-
**Female**	11	Negative	Negative	Unidentified	BWT + PVRUV > 15 cc	Small sized RK + Scar in LK	Bilateral grade 4 VUR	Stage 3 of renal scar in RK	OAB+ detrusor hyper activity
**Female**	5.5	Negative	Positive (pyelonephritis)	Unidentified	BWT	Normal	Bilateral grade 3 VUR + trabeculated bladder with diverticulum +small sized bladder	3 of renal scar in LK	-
**Female**	8	Constipation, encopresis	Positive (pyelonephritis)	Unidentified	BWT	Bilateral hydro nephrosis	Bilateral grade 5 VUR	Stage 2 of renal scar in RK	-
**Female**	9	Encopresis	Positive (cystitis)	Unidentified	BWT + PVRUV > 15 cc	Small sized LK	grade1 VUR in LK + trabeculated bladder	Was not performed	-
**Female**	4 y and 10 mo	Encopresis	Negative	Positive	BWT	Normal	grade3 VUR in LK + trabeculated bladder + Spinning top deformity	Normal	Normal uroflowmetry
**Female**	7	Negative	Negative	Negative	Trabeculated bladder	Left hydro nephrosis	Grade 3 VUR in RK	Normal	OAB
**Male**	4 y 10 mo	Negative	Negative	Positive	BWT	Normal	Grade 5 VUR in RK^ a^	Normal	OAB + detrusor hyper activity
**Female**	6	Negative	Positive (cystitis)	Unidentified	BWT	Small sized RK + Bilateral mild hydro nephrosis	Grade 3 VUR in RK and grade I in LK^ a^	Stage 3 of renal scar in RK, Stage 2 of renal scar in LK^ a^	-
**Female**	4 y and 4 mo	Negative	Negative	Unidentified	PVRUV > 15cc	Normal	Grade 3 VUR in LK	Normal	-
**Female**	4.5	Constipation, encopresis	Negative	Unidentified	BWT	Small sized kidneys + dilated left ureter	Grade 4 VUR in LK + trabeculated and vertical bladder	Stage 2 of renal scar in LK	-
**Female**	10 y 3 mo	Negative	Negative	Positive	BWT + PVRUV > 15 cc	Normal	Grade 4 VUR in RK	Normal	Normal
**Female**	4 y and 10 mo	Encopresis	Positive (pyelonephritis)	Positive	-	-	Grade 3 VUR in RK and grade 4 in LK	Stage 2 of renal scar in RK	-
**Female**	7 y and 10 mo	Encopresis	Negative	Negative	BWT + widening of bladder neck	Normal	Grade 3 VUR in RK	-	-
**Female**	4.5	Constipation	Positive (pyelonephritis)	Positive	Normal	Normal	grade 2 VUR in LK + widening of bladder neck	Bilateral stage 2 of renal scar	OAB

^a^ Abbreviations: BWT, bladder wall thickness; OAB, overactive bladder; RK, Right Kidney; LK, Left Kidney; PVRUV, post voiding residual urinary volume; UDS, Urodynamic studies; US, ultra-sonography; UTI, urinary tract infection; VUR, mainly vesicoureteral reflux.

## 5. Discussion

By reviewing the literature, we did not find any investigation in children with physiological UI to define LUT urological abnormalities. Therefore, we decided to conduct a study in a group of children with physiological UI according to the standard definition. According to our local pediatric hemodialysis centers registries, the most common etiology of end stage renal diseases is still VUR (24-28). Our clinical practice (non-published) suggested that LUT symptoms and incontinence were the second most common symptoms in children with VUR. In addition the previous studies in subjects with NMNE revealed that those with daytime UI were at greater risk of VUR (29, 30).

Literature review and comments by Oppel et al. (31) and Bernard-Bonnin et al. (32) persuaded us for doing the study. Oppel et al. believed that as most children achieve daytime control of their bladders by four years of age, diurnal enuresis should be considered abnormal in any child older than four years of age who wets daily either primarily or after a period of successful toilet training (31). In addition, Bernard-Bonnin et al. suggested renal and bladder US in all children older than four years of age with persistent diurnal enuresis. They believed that VCUG should be performed in cases of UTI to detect VUR or urethral obstruction (32). Whereas, others believed that incontinence was usually functional in children and there were rarely underlying anatomical or neurological diseases; hence, invasive studies would not be required (1, 8, 9), and diagnostic studies should be reserved for those with evidence of a structural or neurologic abnormality or associated urinary tract symptoms such as infection or hematuria (8). Reflux is usually congenital, occurs in families, and affects approximately 1% of children (33). The incidence of VUR in our series (both groups) was significantly higher than the general population. Different studies determined a wide normal range for urinary control during the day (1-7). Blum et al. (34) noted that early initiation of intensive toilet training correlates with an earlier age in the maturation of this process, but a longer time of toilet training at the age of 18 to24 months. Hoebeke's et al. recommended a detailed and structured history taking, voiding diary, and a thorough physical examination as first evaluation steps followed by urinalysis, uroflowmetry with pelvic floor EMG, and adding the pre- and post voiding bladder US (35). The International Continence Society (ICS) defined dysfunctional voiding as intermittent and/or fluctuating flow rate due to involuntary intermittent contractions of the periurethral striated muscle during voiding in neurologically normal individuals (36). It is suggested that the most common mechanism underlying pediatric daytime incontinence is detrusor overactivity; a voiding pattern similar to that is observed in infantile period (37, 38). In our series, abnormal urodynamic findings were reported in 62.5% and 63.6% of children younger and older than five years of age, respectively (P > 0.05). EMG and CMG were performed in special patients (those with abnormal uroflowmetry, increased BWT, or PVURV > 15 mL); thus, real incidence of voiding dysfunction in our cases might not be as high as we found by investigating these patients. Small sample size, especially in group 2, was another limitation to our study which should be considered while interpreting the results of UDS. Although, detrusor over activity is reported as the most common mechanism underlying pediatric daytime incontinence (38) we found OAB and detrusor over -activity (hyperactivity) as main types of dysfunctional voiding in our series, which were reported in 16 (53.3%) and 15 (50%) patients, respectively. Detrusor over activity accompanied by OAB in 12 (40%) patients (Table 3). 

An attractive topic in pediatric UI and voiding dysfunction is applying pediatric lower urinary tract scoring system (PLUTSS) which is a standardized questionnaire used to screen children with bladder dysfunction and to follow up their response to treatment (38). Different researchers used this scoring system to predict voiding dysfunction in children (39, 40).We did not use this system of scoring in our study since definition of LUT symptoms are just applicable for children older than five years of age (12).

What are the normal uroflowmetry curves in children? Chang et al. (41) evaluated uroflowmetry curves in 190 healthy kindergarten children with mean age of 4.5 years. Among them, 154 (81%) children had bell-shaped (normal curve), while 36 (19%) children were diagnosed as having abnormal curves including one interrupted, 24 plateaus, 11 staccato, and no tower-shaped curves. We found abnormal curves in 14 (58.3%) out of 24 participants in group 1 and 6 (54.5%) out of 11 children in group 2. Children with daytime UI are at higher risk to develop UTI (42). In our study, 17 (one-third of cases) and 6 (40%) patients in groups 1 and 2 had UTI at first evaluation, respectively. In children with daytime incontinence and recurrent symptomatic UTI or abnormal US findings such as double systems, ureterocele, or upper tract dilatation, VCUG can give additional information on VUR and bladder outlet or urethral anomalies (35). Ultrasonography is used to detect renal abnormalities (dilated pelvicalyceal system, duplex kidney, and reduced renal parenchyma), pathological amounts of residual urine (43), and thickening of the bladder wall, which is indicative of a bladder voiding disorder (1). Kidney-bladder US is a non-invasive method for screening urological anomalies. In our study, results of US were not helpful to differentiate those at greater risk of VUR. About 50% of patients with VUR had normal Kidney US including four children younger than five years of age. Normal Kidney US was reported in one, four, and two patients with mild, moderate, and severe grades of VUR, respectively (Table 4). 

Since association of intermittent incontinence and urological anomalies is rarely reported (1), we expected to find urological anomalies in a few children. It was unexpected that VUR was found more frequently in children younger than five years of age (P = 0.051). We supposed that as children were evaluated in a tertiary referral center, younger children (< 5 years of age) with more severe incontinence were referred for evaluation; however, this fact should be accepted that even if they presented with severe form of the disease, the form of incontinence was intermittent instead of continuous. This finding suggests that some cases of intermittent incontinence in children aged four to five years might be pathologic and all of these children should not be considered as physiological UI even when physical examination reveals normal findings.

Bernard-Bonnin et al. (32) recommended doing VCUG in children older than four years of age who have daytime incontinence in case of UTI, while in our cases nine out of 15 children with VUR did not have any previous history of UTI. In our subjects, the history of UTI was uncommon in children with physiological UI who had VUR, and just two out of seven patients had a previous history of infection (Table 4). It seems that history of UTI alone is not a sensitive marker for defining cases who need to be screened for VUR. Constipation is common in children, affecting 0.7% to 29.6% of the general population worldwide (38, 44), and is frequently associated with fecal incontinence (36). Many reports suggested correlation between constipation and functional bladder-outlet discoordination, which is responsible for UI, UTI, VUR, and even false uroradiologic pathology in constipated patients (35, 44-46). In our series, constipation was a common finding; 31.3% of children with physiological UI and 21.6% of patients with NMNE had constipation.

Our study suggests that intermittent daytime UI in children younger than five years of age should not always be considered as a physiologic phenomenon. In addition, it seems that VUR and abnormal urodynamic findings are as common in children with physiological UI as those with NMNE who wetted during the day. Whether LUT urological anomalies such as VUR and abnormal urodynamic findings are common in children considered as physiological UI, is a question which should be answered by reviewing larger patients’ groups and emphasizing the role of different variables such as findings on physical examination, severity of UI, history of UTI, age of onset of toilet training, and associated bowel symptoms. This study supported the results of our previous studies (29, 30) that suggested screening for VUR in case of bed-wetting associated with UI. Although, our previous studies were performed on children with enuresis (age group > 5 years), it might be true for some cases of physiological UI.
